# Primary biliary cirrhosis

**DOI:** 10.1186/1750-1172-3-1

**Published:** 2008-01-23

**Authors:** Teru Kumagi, E Jenny Heathcote

**Affiliations:** 1 Department of Medicine, Toronto Western Hospital (University Health Network/University of Toronto), Toronto, Ontario, Canada

## Abstract

Primary biliary cirrhosis (PBC) is a chronic and slowly progressive cholestatic liver disease of autoimmune etiology characterized by injury of the intrahepatic bile ducts that may eventually lead to liver failure. Affected individuals are usually in their fifth to seventh decades of life at time of diagnosis, and 90% are women. Annual incidence is estimated between 0.7 and 49 cases per million-population and prevalence between 6.7 and 940 cases per million-population (depending on age and sex). The majority of patients are asymptomatic at diagnosis, however, some patients present with symptoms of fatigue and/or pruritus. Patients may even present with ascites, hepatic encephalopathy and/or esophageal variceal hemorrhage. PBC is associated with other autoimmune diseases such as Sjogren's syndrome, scleroderma, Raynaud's phenomenon and CREST syndrome and is regarded as an organ specific autoimmune disease. Genetic susceptibility as a predisposing factor for PBC has been suggested. Environmental factors may have potential causative role (infection, chemicals, smoking). Diagnosis is based on a combination of clinical features, abnormal liver biochemical pattern in a cholestatic picture persisting for more than six months and presence of detectable antimitochondrial antibodies (AMA) in serum. All AMA negative patients with cholestatic liver disease should be carefully evaluated with cholangiography and liver biopsy. Ursodeoxycholic acid (UDCA) is the only currently known medication that can slow the disease progression. Patients, particularly those who start UDCA treatment at early-stage disease and who respond in terms of improvement of the liver biochemistry, have a good prognosis. Liver transplantation is usually an option for patients with liver failure and the outcome is 70% survival at 7 years. Recently, animal models have been discovered that may provide a new insight into the pathogenesis of this disease and facilitate appreciation for novel treatment in PBC.

## Disease name

Primary biliary cirrhosis (PBC)

## Definition/diagnostic criteria

Primary biliary cirrhosis (PBC) is a chronic, slowly progressive, autoimmune, cholestatic liver disease that affects predominantly middle-aged women [1]. Diagnosis can be typically established by the triad: antimitochondrial antibodies (AMA) in serum, cholestatic indices and liver histology diagnostic or compatible with PBC.

Addison and Gull have first described PBC in 1851 [2]. The label 'Primary biliary cirrhosis' was adopted in 1949, even though not all patients were cirrhotic at diagnosis [3]. The description of 'Chronic non-suppurative destructive cholangitis' [4], a more appropriate term for the disease, suffers from being too long and thus has never been adopted.

## Epidemiology

Patients usually present in the fifth to seventh decade and PBC is rarely diagnosed in teenagers [5]. PBC have a female predominance with an 8:1 female-to-male ratio [6]. PBC affects individuals of all ethnic origin and accounts for 0.6~2.0% of deaths from cirrhosis worldwide [7]. Its prevalence is estimated to be between 6.7 and 940 cases per million-population (the latter in women >40 yrs old in United Kingdom), while its incidence is estimated to be between 0.7 and 49 cases per million-population per year [8-15]. The highest incidence and prevalence rates come from the United Kingdom [8,15], Scandinavia [9], Canada [10,11] and the United States [12,13], all in the northern hemisphere, whereas the lowest is from Australia [14]. There is no clear worldwide evidence to support the concept of "a polar-equatorial gradient" as it has been reported for other autoimmune conditions [16], but it may also be the case in PBC.

## Clinical description and diagnostic methods

The diagnosis of PBC is based on a combination of clinical features, an abnormal liver biochemical pattern (a cholestatic picture with or without a hepatitis picture) persisting for more than six months and the presence of detectable AMA in serum. The diagnosis may be confirmed by finding characteristic histological features. A "probable" diagnosis requires the presence of two of these three criteria, and a "definite" diagnosis requires all three. The diagnosis of PBC is now made more often and earlier in the course of the disease than it used to be [17,18], likely due to the widespread use of AMA testing and the performance of biochemical screening in healthy individuals [19]. AMA is negative in approximately 10% of patients who otherwise have all the features typical of PBC [20]. All AMA negative patients with cholestatic liver disease should be carefully evaluated for the presence of PBC by cholangiography as well as liver biopsy.

### Clinical features

PBC is a chronic liver disease generally characterized by a slow progression but a highly variable clinical course. More than half of patients diagnosed today are asymptomatic at diagnosis [21,22]. They are generally identified by finding of an elevation of only serum alkaline phosphatase (ALP) or/and total serum cholesterol, often by chance through routine check up. The diagnosis may also be made in patients undergoing further investigation for an associated autoimmune disease such as scleroderma. It may take years before asymptomatic subjects develop symptoms of the disease. But asymptomatic disease is not synonymous with early liver disease. Little is known about why some patients remain asymptomatic and other progress rapidly and develop liver failure. The severity of the liver disease may be discordant with the severity of symptoms. In symptomatic patients, fatigue and pruritus are the most common presenting complaints [10]. Patients with cirrhosis may present with ascites, hepatic encephalopathy and/or esophageal variceal hemorrhage. However, variceal hemorrhage is not limited to those with cirrhosis, as presinusoidal portal hypertension due to nodular regenerative hyperplasia (NRH) of the liver is sometimes present [23]. Once jaundice is present, fat-soluble vitamin deficiency and malabsorption (*e.g.*: steatorrhea and weight loss) may occur.

The prevalence of fatigue has been reported to be as high as 80% [24]. Fatigue does not correlate with the liver disease severity [25] and liver transplantation does not improve fatigue [26].

Pruritus, when present, may be a debilitating symptom that significantly impairs quality of life in PBC, just as may fatigue. Usually, pruritus occurs months to years earlier than jaundice, and may improve with progression of liver disease! It is usually less severe in sunny weather and, being worse at night, it may interfere with sleep. Severe pruritus causes sleep deprivation and may even lead to suicidal ideation [27]. Sometimes PBC is initially diagnosed during pregnancy prompted by the symptom of pruritus that fails to disappear in the post partum period, unlike intrahepatic cholestasis of pregnancy.

Approximately 10% of patients complain of vague intermittent right upper quadrant pain or discomfort, which usually resolves spontaneously [28].

### Physical examination

Physical examination may be normal in those with early-stage disease. As the disease progresses, patients may develop signs of portal hypertension such as splenomegaly and collateral vessels, and features of cholestasis such as skin pigmentation due to increased melanin deposition in the skin. In late stage disease, when both liver and spleen are enlarged, signs of liver failure such as ascites and encephalopathy are seen [22]. However, there is no specific dermatological feature in PBC patients with pruritus other than diffuse pigmentation and scratch marks. Xanthelasma are seen in a minority of patients and xanthomas are rare nowadays, despite PBC related hypercholesterolemia being present. Signs and symptoms of scleroderma, particularly affecting the hands and swallowing, may be present.

### Laboratory tests

Serum ALP and gamma-glutamyl transpeptidase (GGT) are raised and serum aminotransferase can also be elevated [29]. Serum AMA are detected in more than 90% of affected individuals [20]. Elevated levels of immunoglobulin M are common in PBC [30,31]. Titer of AMA does not carry any clinical significance nor do AMA profiles [32]. Although rare findings in patients with PBC, "nuclear-rim" and "multiple nuclear-dot" patterns are highly specific for PBC irrespective of AMA status, and may provide supportive evidence for PBC especially in those who test AMA negative [33]. Liver synthetic function, *i.e.*: changes in albumin and prothrombin time (PT) are observed with disease progression. But hyperbilirubinemia may be present earlier due to the cholestatic nature of this disease.

### Imaging

Lymphadenopathy in the abdomen is seen in 80% of patients with PBC [34]. There is no specific feature on abdominal images in PBC although the intraabdominal lymph nodes may be so large as to suggest lymphoma. Abdominal images are not necessary to confirm the diagnosis of PBC in patients who test positive for AMA. However, those patients who are AMA negative and thought to nevertheless have PBC need radiographic demonstration of a normal biliary tree on cholangiography, either by magnetic resonance cholangiopancreatography (MRCP) or endoscopic retrograde cholangiopancreatography (ERCP), to exclude other possible diagnoses, particularly primary sclerosing cholangitis (PSC). Findings from abdominal images in advanced PBC often resemble those seen in other forms of cirrhosis, with a heterogeneously attenuating liver, varices and splenomegaly.

### Histology

**Figure 1:**
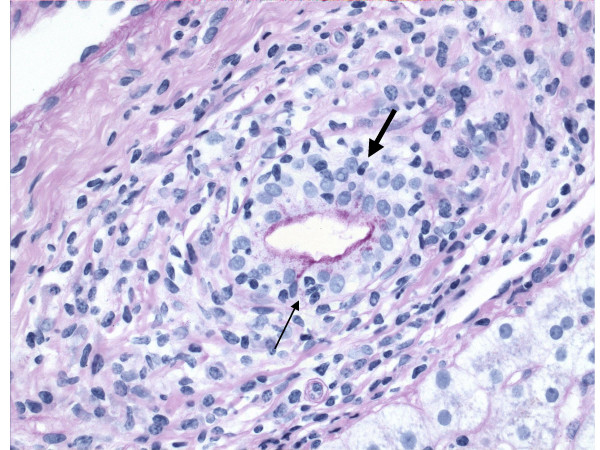
Primary biliary cirrhosis, demonstrating chronic non-suppurative destructive cholangitis (stage 1 of Scheuer's classification). Infiltration of lymphocyte (*arrow*) and plasma cell (*bold arrow*) into bile duct is shown. (D-PAS.)

**Figure 2:**
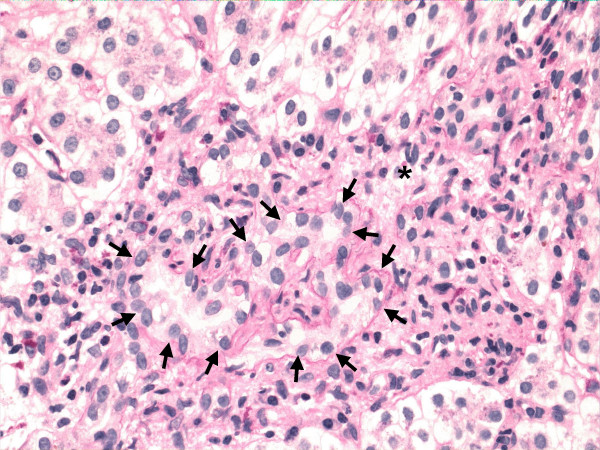
Primary biliary cirrhosis, showing ductular proliferation (stage 2 of Scheuer's classification). Small ductular structures (*arrow*) is shown in a portal tract (*asterisk*). (Hematoxylin-eosin.)

The characteristic lesion of PBC is the asymmetric destruction of the intralobular bile ducts within portal triads. Histological staging systems of PBC have been proposed and they have been divided into 3 or 4 distinct stages, according to the degree of fibrosis, inflammation and/or bile duct damage [4,35-37]. According to the Ludwig's classification [37], stage 1 is defined by portal inflammation. Stage 2 is extension of this inflammation beyond portal tracts into the surrounding parenchyma, with or without associated duct loss. In stage 3, fibrous septa link adjacent portal triads. Stage 4 represents cirrhosis. On the other hand, with Scheuer's system [35], stage 1 is referred to as the florid duct lesion or chronic non-suppurative destructive cholangitis (Figure 1). In stage 2, there is proliferation of the small bile ductules (Figure 2). Stage 3 is characterized by fibrosis or scarring. Stage 4 is cirrhosis. However, the entire liver is not always uniformly involved, and a single biopsy may demonstrate the feature of all four stages simultaneously. Thus both under- and over-estimation may occur. It is crucial to have a sufficient size of specimen to minimize error [38]. There should be a minimum of 10 portal tracts visualized before a confident diagnosis of bile duct loss can be established. Although liver biopsy is not always necessary to make a diagnosis of PBC, it does allow the severity of disease to be clarified. It also may indicate the need of specific therapeutic regimens or management (such as ultrasound screening for hepatocellular carcinoma, HCC, in PBC patients found to be cirrhotic) and, additionally, may be used as an objective way for evaluating the response to treatment.

### Associated diseases (Table 1)

**Table 1:** Diseases or disorders associated with PBC

Sjögren's syndrome (Sicca syndrome)	
Scleroderma	
Raynaud's disease	
CREST syndrome	
	Calcinosis cutis
	Raynaud's phenomenon
	Esophageal dysmotility
	Sclerodactyly
	Telangiectases
Systemic lupus erythematosus	
Rheumatoid arthritis	
Mixed connective tissue disease	
Polymyositis	
Cutaneous disorders	
	Dermatomyositis
	Lichen planus
	Pemphigoid
	Psoriasis
Autoimmune thrombocytopenic purpura	
Pernicious anemia	
Sarcoidosis	
Myasthenia gravis	
Autoimmune thyroid disease	
	Chronic thyroiditis (Hashimoto's disease)
	Grave's disease (Basedow's disease)
Diabetes Type 1 (Insulin-dependent diabetes mellitus)	
Addison's disease	
Inflammatory bowel diseases	
	Celiac disease
	Ulcerative colitis
	Crohn's disease
Gallstones	
Pulmonary fibrosis	
Glomerulonephritis	

Associations between PBC and other autoimmune diseases have been reported. In an older report of extrahepatic autoimmune diseases among patients with PBC, 84% and 41% of the patients had at least one and more than two associated autoimmune disease, respectively, in addition to PBC. Sjögren syndrome, including sicca syndrome was the most commonly associated autoimmune disease, seen in 66% of the patients [39]. However, this report was not a population-based study, thus, their true extent and pattern was unknown. Recently, a population-based cohort study has shown the greatest relative increase in disease prevalence for scleroderma (8% of patients); 53% of patients had at least one associated autoimmune disease. The risk of Sjögren syndrome and Raynaud's phenomenon was about 4-fold higher than unaffected controls. The significance of systemic lupus erythematosus (SLE) as a risk factor for PBC was also confirmed. In this report, AMA negative patients had a significantly higher prevalence of additional autoimmune condition than AMA positive patients (79% *vs. *49%) [40]. In patients with PBC and/or CREST syndrome, overrepresentation of a T-cell receptor beta chain variable region (TCRBV3) was documented, suggesting a distinct disorder [41]. These associations further support the hypothesis of genetic susceptibility as a predisposing factor for PBC.

### Differential diagnosis (Figure 3)

**Figure 3:**
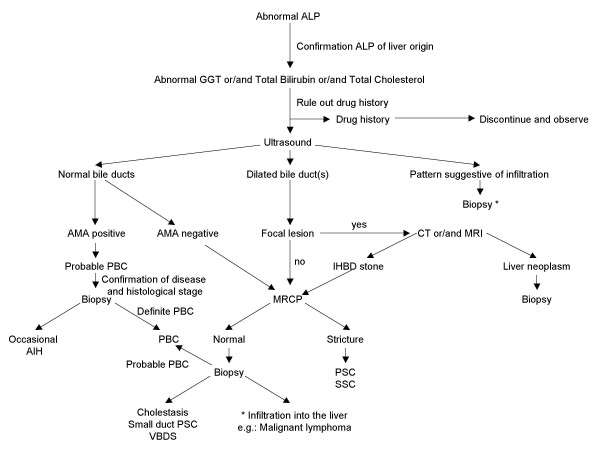
Diagnosis of PBC. Abbreviations: AIH, autoimmune hepatitis; ALP, alkaline phosphatase; AMA, antimitochondrial antibody; CT, computed tomography; GGT, gamma-glutamyl transpeptidase; IHBD, intrahepatic bile duct; MRCP, magnetic resonance cholangiopancreatography; MRI, magnetic resonance imaging; PBC, primary biliary cirrhosis; PSC, primary sclerosing cholangitis; SSC, secondary sclerosing cholangitis; VBDS, vanishing bile duct syndrome.

In 2007, the majority of patients with PBC present without symptoms, most are identified with abnormal liver enzymes at the time of a regular check up or during work up for another disease. It is not difficult to diagnose PBC when the patients have typical features, particularly AMA positivity. However, 0.5% of people in general population are also positive for AMA [42]. As obesity and non-alcoholic fatty liver disease (NAFLD) is becoming a serious issue worldwide, abnormal liver biochemical tests are not unusual in the general population. These factors all have to be taken into account in the differential diagnosis, especially when patients with abnormal liver enzymes are of a 'cholestatic nature', not unusual in NAFLD or drug induced liver injury.

All AMA negative patients with cholestatic liver disease should be carefully evaluated. In the differential diagnosis, the initial step is to clarify if the site of the damage is extra-hepatic, intra-hepatic or both. In addition to a carefully taken history, ultrasound (US) may help to differentiate these conditions. If abnormalities of the biliary tree are seen on US, cholangiography (MRCP) will help further differentiation, particularly in the diagnosis of PSC. The typical bile duct irregularities of PSC are demonstrated on cholangiography, but in early PSC as well as small duct PSC, the biliary tree may appear normal. Cholangiography may also help in the diagnosis of other bile duct lesions such as stones, cyst or tumor that may be not visualized on US [43]. If the extra-hepatic biliary system appears normal, then liver biopsy is required. Liver biopsy allows differentiation between cholestatic disease (generally a "vanishing bile duct syndrome") and an infiltration in the liver, *i.e.*: lymphoma.

Secondary biliary cirrhosis is a biliary cirrhosis that develops secondary to a pathogenic process or injury of known cause (just as is the case for secondary sclerosing cholangitis [44]) and may occur with chronic biliary obstruction of any cause (*eg*. hepatolithiasis or cystic fibrosis) [45].

It is also important to note that a possible change in diagnosis should continue to be considered during follow-up as well as at the time of initial diagnosis. Fatty liver and drug induced liver injury are the most common comorbidities that may "interfere" with the initial diagnosis or during follow up. These should be always considered if a patient with PBC has a sudden change in the liver enzyme pattern. Autoimmune hepatitis (AIH) is another disease that may develop subsequently [46], thus, it also should be considered in the differential diagnosis, particularly with a transaminitis. The most reliable feature to suggest development of AIH would be a further rise in serum IgG. Similarly, a co-existent untreated celiac disease (present in 7% of patients with PBC, particularly in Caucasians) may cause an increase in liver enzymes or a failure to normalize with UDCA [47]. A recent general population-based study from Sweden has shown that odds ratio of PBC and fatty liver in celiac disease is 15.0 and 5.83, respectively [48]. The anti-transglutaminase antibody test is specific and highly diagnostic for celiac disease. There are reports of improvement of liver enzymes or even reversal of hepatic failure after introducing gluten free diet in patients with PBC associated with celiac disease [49]. Thyrotoxicosis may lead to the sudden onset of jaundice [50].

## Etiology

### Genetic factors

Familial predisposition to PBC is not rare, its prevalence rate among first-degree relatives has been estimated to be approximately 5% [51], which is more common than in unrelated persons. It has been reported that familial PBC is related to maternally inherited factors, and that the disease tends to present earlier in the second generation [52], and is most common in mother-daughter and sister-sister combinations [53]. Familial cohort studies have shown that the concordance rate of PBC in monozygotic twins was 63% *versus *0% among dizygotic twins. Among monozygotic twins, the age of disease onset was similar, but there were differences in the natural history and disease severity, emphasizing the role of epigenetic factors or simply environmental factors [54]. Although the disease is virtually identical in women and men, recent data suggest that X-chromosome monosomy in lymphoid cells is common in women with PBC [55]. Genes governing immune tolerance are carried on the X gene. Case reports of PBC in patients with Turner syndrome also support the possible role of genes on the X chromosome in the genetic predisposition to PBC [56].

### Infection

Several environmental factors have been suggested as potential causative initial agents, particularly bacteria. *Escherichia coli *and their "rough" mutant have been reported in excess in the stools of patients with PBC; in addition, the incidence of urinary tract infections is high in PBC patients [57,58]. Recently, *Novosphingobium aromaticivorans*, a ubiquitous organism that metabolizes organic compounds and estrogens, has been proposed as a candidate for the induction of PBC [59]. The titers of antibody against the lipoylated bacterial proteins of *N. aromaticivorans *were 1,000-fold higher than those to *E. coli*, in patients with PBC including patients in early stage of disease.

Various other bacteria, such as *Chlamydia pneumoniae, Helicobacter pylori *and *Mycobacterium gordonae *have also been implicated as putative pathogens [60-63]. However, no compelling data have been provided to show that any individual agent can be reproducibly detected in the liver of patients with PBC [64].

Recently, it has been reported that human betaretrovirus (that shares marked genetic homology with the mouse mammary tumor virus) infection may be detected in approximately 75% of patients with PBC (by reverse transcription polymerase chain reaction and immunochemistry) [65]. This has been supported by histological and biochemical endpoints achieved in a pilot study of an antiretroviral agent [66]. However, other studies have not been able to reproduce these data [67].

### Past surgery

Surgeries including appendectomy, other abdominal surgeries and tonsillectomy are significantly increased in patients with PBC in an epidemiological study in North America [68]. However, an earlier population-based case-control study conducted in England did not show any association with surgical procedures [69]. More recently, a case-control study provided evidence that there was no association between PBC and the occurrence of appendectomy, and pointed out the selection bias present in the previous study done in North America [70]. However, the observation of a PBC-specific immune response to the highly conserved caseinolytic protease P of *Yersinia enterocolitica *in 40% of patients with PBC may support the link with past appendenctomy [71], where infection with *Y. enterocolitica *is one of the major causes of acute terminal ileitis mimicking acute appendicitis [72].

### Environmental chemicals

A recent experiment demonstrated that circulating antibodies isolated from the serum of PBC patients recognized artificially altered (by environmental chemicals) the lipoic acid component of the pyruvate dehydrogenase complex (PDC) epitope [73]. This same group of investigators has shown that 2-octynoic acid, which is widely used in the manufacture of perfumes, lipstick and many common food flavorings, have the potential to modify E2 subunit of PDC (PDC-E2) *in vivo *[74]. The frequent use of nail polish has been linked to risk of having PBC [75]. Smoking has been demonstrated to accelerate the progression of PBC and to be a risk factor for PBC [75,76], possibly due to exposure to chemicals in cigarette smoke. Apparent clusters of populations and a group of individuals living close to toxic waste or specific reservoirs have been reported to have higher rate of incidence or prevalence of PBC and this suggests toxic waste as a risk factor or trigger of the disease [77-79].

## Pathogenesis

Immune mediated destruction of bile duct epithelial cells is thought to drive the pathogenesis of PBC. Although the exact mechanism underlying the pathogenesis of PBC remains uncertain, PBC is regarded as an organ specific autoimmune disease. The hallmark of autoimmune process is the detection of AMA in serum, although a recent study indicates that AMA are detectable, absent transiently in fulminant liver failure [80]. AMA targets three components of M2 family of mitochondrial antigens: the PDC-E2, the 2-oxoglutarate dehydrogenase complex (OGDC) and branched-chain 2 oxo-acid dehydrogenase complex (BCOADC). Of these three, the major autoepitope recognized by both T and B cells is the inner lipoyl domain of the PDC-E2 [81]. PDC is a member of the 2-oxo-acid dehydrogenase family of multi-enzyme complexes and is associated with both cellular and humoral immune responses in PBC.

Autoantibodies to nuclear antigens can be detected in the serum in approximately 50% of patients with PBC, who often have AMA. These antigens include gp210, p62 and lamin B receptor that are nuclear pore proteins, and sp100 that is nuclear body protein. It has been shown that individuals who have sustained antibody response to gp210 antigen in the sera [82] or a high expression of gp210 antigen in small bile ducts in liver tissue [83], are at higher risk of progression to end-stage hepatic failure.

It remains a mystery how PDC-E2 and other epitopes localized to the inner membrane of mitochondria become a targets of autoimmune injury in such highly selective biliary epithelial cells and salivary duct cells [84]. It has been suggested that apoptosis can increase the exposure of PDC-E2 to the immune system, leading to an autoimmune attack. It has been postulated that apoptotic cholangiocytes, unlike other apoptotic cell types, are a potential source of immunogenic PDC-E2 in patients with PBC [85]. Sera from patients with PBC have been found to have antibodies to environmental toxins thought sufficient to break self-tolerance [74]. Down regulation of the anti-apoptotic protein, bcl-2 family, in injured portal tracts has been demonstrated to be a marker of ongoing apoptosis of cholangiocytes [85,86].

Patients with PBC are usually characterized by the presence of PDC-E2-specific CD4 and CD8 positive T cells in the sera [87] and PDC-specific T cells in the liver [88], mostly during early phase of the disease. Recently, a CD8 positive T cell epitope of PDC-E2 (amino acid 159–167), has been identified [89] and HLADR4*0101-restricted CD8 positive T cells from livers of patients with PBC have demonstrated cytotoxicity against PDC-E2 (amino acid 159–167) pulsed autologous cells [90]. These findings strongly suggest a contribution by T-cell mitochondrial responses in the bile duct injury of PBC.

A mouse model of NOD.c3c4 mice congenitally derived from a non-obese diabetic strain recently described develops an autoimmune biliary disease and tests AMA positive [91]. Two other animal models, IL-2 receptor alpha (-/-) mice and TGF-beta receptor II dominant-negative mice also show an association with AMA and chronic biliary disease [92,93]. The affected biliary epithelium is infiltrated with CD4 positive and CD8 positive T cells in all of these models. However, granulomas and eosinophilic infiltration are seen only in NOD.c3c4 mice. Whether these mouse models will facilitate understanding the pathogenesis of PBC in human is unknown.

Chronic inflammation and repeated injury to small bile ducts triggers proliferation of bile ductular cells and fibrosis caused by adjacent myofibroblastic cells in the hepatic mesenchyme. Fibro-proliferative bile ductular cells often extend into the hepatic parenchyma, bridging adjacent portal areas and leading to biliary cirrhosis without renewal of bile ducts [94]. The pathogenesis of the fibrogenic response to bile duct injury has been recently investigated with a new concept the "Hedgehog pathway" in the liver, which is a system that regulates the viability and differentiation of various progenitors during embryogenesis [95]. The Hedgehog pathway has recently been shown to promote the growth of both biliary epithelial cells and stromal cells, where signaling between them may modulate the mechanism of bile duct injury [96]. The proximity of the myofibroblasts and cholangiocytes suggests that mesenchymal-epithelial crosstalk promotes the fibro-proliferative response to cholestatic liver injury *via *the Hedgehog pathway [97].

## Treatment for underlying disease

Three groups of drugs have been evaluated in PBC: immunomodulators, antifibrotics and anticholestatics. Currently, the first-line therapy is ursodeoxycholic acid (UDCA), an anticholestatic [98].

### Ursodeoxycholic acid (UDCA)

UDCA is a bile acid, which naturally comprises 2% of human bile. Treatment with UDCA improves serum biomarkers such as bilirubin, ALP, GGT, alanine aminotransferase, aspartate aminotransferase, cholesterol and IgM levels [99-101].

According to the combined data from three controlled trials, UDCA between 13 and 15 mg/kg body weight is considered the preferred treatment for PBC [102]. It is safe and the side effects are few. Some patients suffer from hair thinning, gain in weight and/or diarrhea. Significant weight gain that occurs in the first 12 months of treatment persists for the duration of treatment and occurs independent of baseline body mass index (BMI) [103].

UDCA improves 10-year survival [104] and/or delays the progression of hepatic fibrosis in early-stage disease [105], and the development of esophageal varices in patients with PBC [106]. Recently, the Spanish group has shown that a good biochemical response to UDCA (defined by an alkaline phosphatase decrease greater than 40% of baseline values or to normal levels) within 1 year of starting treatment is associated with a similar survival to a matched Spanish control populations but this is not so for those patients who show no biochemical response following introduction of UDCA [107]. However, these findings are not seen in patients with advanced stage disease at baseline. Non-randomized controlled studies from Greece and the Netherlands indicate that in most patients with PBC (particularly those who are in early stage of disease), the 10-year survival is comparable to that in the general population [108,109].

A recent meta-analysis has shown that long-term treatment with mid-dose UDCA (10–20 mg/kg/day) improves the liver biochemistry and survival free of liver transplantation, and delays histological progression in the early-stage patients [110]. But whether there is a survival benefit of treatment with UDCA in PBC still remains controversial. The most recent meta-analysis from Cochrane group included 6 trials of short duration, some of them non-randomized studies, and there were some data collection errors with varying doses of UDCA. Thus, their results suggesting no survival benefit with UDCA treatment need to be questioned [111].

There is no data indicating that UDCA is teratogenic but if a pregnancy can be planned then it may be wise to stop any medication before and during the first trimester. UDCA is used to treat intrahepatic cholestasis of pregnancy [112] and has not been shown to be toxic to the fetus if given during the second or third trimester.

### Other medications

Budesonide (a glucocorticoid with high receptor activity and high first-pass metabolism in liver) combined with UDCA has been shown to improve liver histology in 2–3 years in a patient population with or without failure of UDCA monotherapy [113,114]. Rautiainen *et al*. have shown that the plasma concentrations of budesonide do not differ when the patients were randomized to receive either budesonide and UDCA, or budesonide alone for 3 years, at least in early PBC [115]. But the combination of budesonide and UDCA has been shown to be associated with decreased bone mass density in the femoral neck and lumbar spine in some patients (probably those with cirrhosis) [116]. This combination therapy is promising but the observation period for side effects is too short at present, thus, studies with longer follow-up using a combination of budesonide and UDCA are necessary to confirm that the benefit outweighs the risks.

Various adjuvant medications have been used alone or in combination with UDCA in PBC, particularly in patients with an incomplete response to UDCA monotherapy. These include systemic corticosteroids [117], azathioprine [118], cyclosporine [119], mycophenolate mofetil [120], methotrexate [121], chlorambucil [122], penicillamine [123], colchicine [124], silymarin [125] and bezafibrate [126]. Some of the studies have shown a benefit in terms of improving biochemical markers, decreasing the intensity of pruritus, improving liver histology, reducing the incidence of major complications of cirrhosis and delaying the need for liver transplantation. However, neither the large trials nor the pilot studies have indicated any survival benefit from these additional agents.

### Liver transplantation

PBC used to be a common indication for liver transplantation in adults. However, the proportion of patients with PBC who undergo liver transplantation has recently decreased, most probably because of the marked increase of transplantation for cirrhosis due to viral hepatitis, alcoholic liver disease and HCC. The assessment for liver transplantation in PBC is indicated when the patients show features of end-stage liver failure such as profound jaundice with or without intractable ascites, episodes of spontaneous bacterial peritonitis, and/or encephalopathy resistant to appropriate treatment, *i.e. *situations with expected survival less than 1 year. The Mayo Risk Score, which calculates 2-year survival rate is useful in this regard. Even though this calculation was developed before UDCA treatment was introduced, it appears valid even when UDCA is used [127].

Recently, the MELD (model for end-stage liver disease) score has been found helpful to decide organ allocation of patients who are already on the waiting list. The score includes bilirubin, creatinine and INR (international normalized ratio). The height of the serum bilirubin level alone is a very important factor when considering the appropriate timing of referral for liver transplantation assessment for patients with PBC. One study has shown that patients should be referred when their serum bilirubin level reaches to 100 **μ**mol/l (5.9 mg/dl) and that the outcome post transplantation is better than without transplantation when the serum bilirubin level is less than 170 **μ**mol/l (10 mg/dl) [128]. The 5-year survival rate after orthotopic liver transplantation (OLT) in PBC may be as high as 85%, one of the best outcomes of any liver diseases.

PBC recurs post-OLT in about 20% of patients, usually between 3 and 7 years post-OLT [129-131]. There is no correlation between AMA titer and clinical or histological recurrence of PBC post-OLT. The hallmark feature of recurrence is granulomatous bile duct destruction seen on liver biopsy. A plasma cell infiltrate also characterizes recurrent PBC and distinguishes it from other processes such as drug induced liver injury and acute or chronic rejection [132]. Optimal immunosuppression needs to be provided to prevent recurrence of PBC. It is still controversial as to whether to use cyclosporine or tacrolimus [131,132]. Disease progression after recurrence is slow and retransplantation is rarely necessary. However, there is a report of graft failure due to recurrent disease [133].

## Treatment for overlap syndrome between PBC and autoimmune hepatitis (AIH)

It has been reported that 12–20% of patients have "overlapping" features of PBC and AIH [134,135]. These features may present simultaneously but sometimes features of AIH develop acutely and change the overall pattern of the liver disease [46]. Corticosteroids are usually contraindicated in PBC because they enhance osteoporosis [117], though their use is advocated by some authors for these patients. Features of AIH in PBC may be transient and response to UDCA therapy similar to patients with PBC without features of AIH. [136]. However, a recent pilot study of a long-term outcome suggests that combination of UDCA and corticosteroids may be the best therapeutic option for a strictly defined PBC-AIH overlap syndrome [137].

Patients with AMA negative PBC often test positive for high titer ANA and have higher levels of IgG [138] but liver histology indicates no evidence of features of AIH. However, a small study of HLA typing in AMA positive and negative PBC indicated a lack of DR8 in those AMA negative cases [139].

## Complications of PBC and their management (Table 2, 3)

**Table 2:** Treatment of the symptoms of PBC

Pruritus	1st line	Cholestyramine 4 g/d (before + after breakfast)
	2nd line	Rifampin 150 mg bid
	3rd line	Sertraline (anti-depressant)
	4th line	Naloxone, by an experienced physician
	5th line	Liver transplantation
	Supportive	UV light, Sunlight
	Emergency	Plasmapheresis
Raynauds	1st line	Ca channel blockers
	2nd line	Alternative: prostacyclin and its derivatives, endothelin receptor antagonists and phosphodiesterase inhibitors
Sicca syndrome	Dry eyes	Artificial tears
	Dry mouth	Dental hygiene, dental visit every 3–6 months
	Dry vagina	Vaginal lubricants

**Table 3:** Screening and treatment of the complications of PBC

		Osteoporosis	Esophageal varices	Hepatocellular carcinoma
Screening	Method	DEXA	Endoscopy	Ultrasound
	Indication	All patients	Cirrhosis, Platelet count <200,000/mm3	Cirrhosis, Portal hypertension
	Interval	Every 2 years	Every 2 years (Every year, if grade 1 varices)	Every 6 months

Treatment	Indication	All patients	>Grade 2 varices	Size, Number, Liver preservation dependant
	Medication	Calcium 1500 mg/d, Vitamin D 1000 IU/d (+ Bisphosphanate, if osteoporotic)	**β**-blockers	
	Others	Dairy products, Exercise, Sunlight	Endoscopic ligation, Endoscopic sclerosing therapy	Radiofrequency ablation, Arterial embolization, Surgical resection, Liver transplantation

### Pruritus

Very recent meta-analysis has shown that there are insufficient data to evaluate the efficacy of cholestyramine [140]. However, the first choice in the treatment of pruritus in cholestatic patients is cholestyramine (4–12 g/d) because it is safe. This agent seems to bind bile that contains the unknown pruritogen of cholestasis in the gastrointestinal tract. Usually it is helpful and effective when taken properly, *i.e. *at the time gallbladder empties (thus it should be taken before and after breakfast). It also has to be taken 4 hours apart from any other medications to avoid their binding. Patients need to appreciate that cholestyramine should be used for long-term prevention of pruritus. It may cause abdominal bloating and constipation.

Rifampin would be the second choice when cholestyramine is not effective, starting at 150 mg twice daily. Usually, its benefit is appreciated in the first week to month of treatment and increasing the dose may help further in those patients who initially fail to respond. A recent meta-analysis indicates that treatment with rifampin leads to complete or partial resolution of pruritus in 77% of patients as compared with 20% treated with placebo or alternative [141]. Rifampin may be considered the first-line therapy for pruritus, but this has not been the case since its efficacy is not widely appreciated, and because of certain untoward side effects and contraindications. Rifampin is an enzyme inducer, hence the potential to cause clinically relevant drug interactions should always be considered, *i.e. *it may inactivate the effect of certain antidepressants. Rifampin may cause hepatitis, hemolytic anemia and renal dysfunction, usually within the initial few weeks of therapy. Patients treated with rifampin need to be monitored closely in this regard.

The precise mechanism of pruritus remains unclear. However, the efficacy of partial external biliary diversion, biliary drainage and plasmapheresis for intractable pruritus in patients with intrahepatic cholestasis suggest that pruritogen(s) such as circulating endogeneous opioids are present in both bile and blood [142-144], and increased levels are reported in patients with PBC [145]. The efficacy of naloxone, an opioid antagonist, for the pruritus of chronic cholestasis supports this hypothesis and may be one of the alternatives when the patients have failed other treatments [146]. Symptoms suggestive of narcotic withdrawal may develop in some patients, thus naloxone should be introduced at very low dose and be built up gradually, ideally by an experienced physician. Attention to the chronic pain syndrome should be paid with long-term use [147].

A recent randomized control trial has shown that sertraline (75–100 mg/day), a serotonine reuptake inhibitor, seems to be an effective and well-tolerated treatment for pruritus caused by chronic cholestasis. This suggests that serotonergic pathways are important in the perception of pruritus [148].

Plasmapheresis should be considered when treatment of pruritus is urgently required, for example when the patient is suicidal due to severe and uncontrollable pruritus. Treatment with ultraviolet light may be beneficial, particularly when the symptom worsens during the winter.

### Fatigue

Fatigue is one of the most common presenting complaints in patients with PBC. There is no known treatment for fatigue in PBC. However, complications such as hypothyroidism, anemia, depression or any other reasons for fatigue have to be carefully excluded rather than assuming PBC to be the cause, particularly as these complications may be treatable. Many drugs such as UDCA [149], cyclosporine [119], thalidomide [150] and antioxidants [151] have been investigated previously but failed to show any efficacy in controlling this debilitating symptom. Even though the cause of fatigue in PBC remains unclear, it may be related to dysfunction of either the corticotrophin-releasing hormone or serotonergic neurotransmitter systems [152]. However, a randomized, controlled crossover trial of ondansetron (a serotonin receptor antagonist) did not confer clinically significant fatigue reduction [153]. In a case series of 8 patients, methotrexate was shown to be efficacious for fatigue in PBC [154]. More recent data suggest a possibility that fatigue is associated with excessive daytime somnolence and altered autonomic function [155,156]. Open label modafinil therapy (a central nervous system stimulant used to treat narcolepsy) was associated with improvement in excessive daytime somnolence and associated fatigue in PBC [157].

### Osteoporosis

Chronic cholestasis is associated with an increased risk of osteopenia and osteoporosis. In the development of osteoporosis in PBC, there are two mechanisms that are commonly used to classify primary osteoporosis: "low-turnover" with normal resorption but reduced synthesis and slow mineralization of matrix and "high-turnover" with increased resorption due to reduced osteoblast function or increased activity of osteoclast [158,159]. Commonly associated risk factors for primary osteoporosis such as postmenopausal status, cigarette smoking and alcohol consumption as well as malabsorption (co-existent celiac disease and/or jaundice) of calcium and vitamin D may be also contributing factors in patients with PBC [160,161]. Inherited predisposition to deficiency of vitamin D associated with particular genetic polymorphisms has been also reported [162]. However, whether patients with PBC are at higher risk of developing osteoporosis than general population is controversial [163,164]. Menopausal status and/or severity of liver disease may be independent factors for osteoporosis development [165,166]. One study does suggest that osteoporosis is a feature of PBC, especially in those patients who are older, thinner and with more advanced liver disease [164]. Recently, it has been confirmed in a population-based study that there is a modest increase in both the absolute fracture risks (12.5 per 1000 person-years for any fracture, and 1.9 per 1000 person-years and 3.4 per 1000 person-years for hip fracture and for ulna/radius fracture, respectively) and the relative fracture risks (approximately 2-fold increased) in patients with PBC, with the excess risk similar in those with more severe disease [167]. Additionally, bone fracture post-OLT occurs due to the combination of preexisting low bone mineral density and early post-OLT bone loss [168]. Therefore, we should consider that all patients with PBC are at risk of osteoporosis, and prophylactic and therapeutic measures need to be undertaken. Screening regularly for bone mineral density using non-invasive, dual energy X-ray absorptiometry (DEXA scan), may identify bone changes prior to bone fractures.

It is difficult to prevent osteoporosis, but both calcium supplements (1500 mg/d) and vitamin D (400–1200 IU/d) are advised. Patients should be encouraged to take exercise on regular basis and be in the sunlight to activate vitamin D. The use of hormone replacement is controversial for postmenopausal patients since it may provide protection against bone loss, but may worsen cholestasis [169]. Recently, alendronate has been demonstrated be more effective than bisphosphonate for the treatment of osteoporosis in PBC [170]. A small pilot trial of vitamin K2 (a fat-soluble vitamin that modulates bone metabolism similarly to vitamin D) has shown the efficacy of vitamin K for treatment of osteoporosis in PBC patients [171].

### Portal hypertension and esophagogastric varices

Nodular regenerative hyperplasia (NRH) is a condition characterized by hepatocytic nodules distributed throughout the liver without perinodular fibrosis and caused by occlusion of small portal veins in the portal tracts. In patients with PBC, NRH may contribute to development of non-cirrhotic portal hypertension [23]. We have shown that patients with PBC and PSC that have a platelet count <200,000/mm^3^, an albumin level <40 g/l, and a bilirubin level >20 micromol/l should be screened for esophageal varices [172]. Once patients are found to have moderate to large esophageal varices, beta-blockers and/or endoscopic variceal ligation (EVL) should be instituted for primary prevention of variceal bleeding [173]. However, nonselective beta-blockers are ineffective in preventing the development of varices in unselected patients with cirrhosis [174]. There is a report showing earlier recurrence of esophageal varices, following endoscopic therapy, in patients with PBC compared with non-PBC patients [175].

### Hepatocellular carcinoma

One large retrospective study has shown that there is an increased risk for development of hepatobiliary malignancies in PBC [176]. Hepatocellular carcinoma (HCC) is the most common malignancy in PBC, particularly in cirrhotic patients. When compared to the rate of HCC in patients with chronic hepatitis C, the relative risks for HCC were similar [177]. Older age, male sex and portal hypertension and/or cirrhosis indicate higher likelihood of HCC [178-180]. Therefore, screening for HCC, for instance, ultrasound at 6 months interval, might be beneficial in these patients.

## Natural history and prognostic considerations

There are numerous reports on the natural history and prognosis of PBC in terms of clinical, biochemical, histological and treatment response. Most of the published data on survival in PBC indicate a poor prognosis but these data were obtained from patients who were diagnosed more than 2–3 decades ago and never received treatment [22]. It appears that, at present, the prognosis is much better than the previously reported, probably because patients are diagnosed at much earlier stage of the disease and treatments are instituted earlier. The features of patients diagnosed with PBC nowadays are different from the days when this disease was first recognized and described. Therefore, it is important that current patients take this into account when they read outdated reports on PBC!

Today, over 60% of the newly diagnosed cases are asymptomatic [22], whereas just a few decades ago most of the patients presented with jaundice. Although most patients are diagnosed and initiated on treatment in the asymptomatic phase, not all patients stay asymptomatic in long-term follow-up. A population-based large cohort study of patients with asymptomatic PBC has calculated the 1-year incidence rate for developing fatigue, pruritus and complications from portal hypertension to be 15%, 13% and 5%, respectively. The majority (80%) of asymptomatic patients become symptomatic within 10 years and the estimates for developing symptoms in 5 and 20 years are 50% and 95%, respectively [29]. Another large cohort of patients with PBC has shown that the median survival or liver transplantation referral from diagnosis was 9.3 years [22]. Patients who were asymptomatic at diagnosis did not live longer than their symptomatic counterparts but the median age of this cohort at baseline was over 60 years (63.1 and 61.8 years in asymptomatic and symptomatic patients, respectively) [29]. Several other reports, based on only a relatively small number of patient deaths during the follow-up period, suggest that the survival of patients asymptomatic at diagnosis falls significantly below than that of gender- and age-matched control groups [181-184]. However, these data are based on patients who had been diagnosed decades ago, before the introduction of UDCA. A substantial proportion of the asymptomatic patients at diagnosis ultimately became symptomatic. The estimated 10-year survival in asymptomatic patients with PBC ranges from 57% to over 90% [14,181,185].

The only report of the natural history of histological changes in PBC (without UDCA but with penicillamine or placebo) has documented that the majority of patients will progress histologically within 2 years. Histological progression to cirrhosis in 4 years was observed in 31%, 50% and 68% of the patients in stage 1, stage 2 and stage 3 (according to Ludwig's classification) at entry, respectively [186]. The incidence of cirrhosis after 5 years of UDCA treatment was 4%, 12%, and 59% among patients followed-up from stages 1, stage 2, and stage 3, respectively [187]. UDCA appears to prevent the progression of the disease, particularly if started in early-stage disease. A recent encouraging report from Spain has shown that in patients with an early-stage disease (stage 1 or stage 2), the biochemical response to UDCA after 1 year is associated with a similar survival to the matched control population [107].

To date, no reliable way has been identified to predict which patients will remain asymptomatic. An older study of asymptomatic patients has suggested that the presence of hepatomegaly, advanced histological stage or the presence of associated autoimmune disorders is strongly predictive of the subsequent development of PBC-related symptoms (pruritus, jaundice, ascites or variceal bleeding) [182,188], while a more recent study has not been able to identify any prognostic variables that would distinguish patients who would become symptomatic from those who would remain asymptomatic [181]. A recent report has suggested that the presence of anti gp210 (one of the antibodies to nuclear antigens) and especially when in high titer may predict those patients who will have a poor outcome [189], but this has been disputed [190]. Another recent study including patients with early-stage disease has demonstrated that the independent predictive factors of cirrhosis development were serum bilirubin (>17 **μ**mol/L), serum albumin (<38 g/L) and moderate to severe lymphocytic piecemeal necrosis [187]. As an environmental factor, smoking has been proven as a contributing risk factor for progression of the disease [76].

Classically, advanced age, elevated INR, jaundice, low serum albumin, edema, ascites and advanced histological stage are strongly correlated with median survival rates of less than 5 years from time of diagnosis in symptomatic patients. Serum total bilirubin is still one of the most reliable predictors for progression of the disease in PBC and it plays a cardinal role in current mathematical models for predicting survival, along with age, serum albumin, PT and severity of edema [191].

The most common cause of death in patients with PBC remains liver failure. Liver-related mortality has been reported as 60% in symptomatic patients [22]. Asymptomatic patients may also eventually die of liver failure (20%) [29]. However, again, these numbers have been retrieved from studies in large tertiary referral centers before UDCA has been available, hence findings may not always reflect current experience. Prospective cohort studies with observation for long periods with serial clinical, biochemical and histological data may provide data that helps to identify marker of disease progression in PBC.

## Future direction

The true significance of UDCA on disease course and natural history remains controversial. Lack of any effect in causing disease regression requires further studies on the pathogenesis of this disease to identify new therapeutic approaches. In this regard, the recent discovery of spontaneous animal models may allow us to better understand the pathogenesis of PBC in humans. Further genetic research may provide a bridge to clinical research, perhaps indicating predictive markers of disease progression as well as facilitating the basis for developing novel approaches to therapy by throwing light on the pathogenesis of PBC. Only these may determine why standard treatments for other autoimmune diseases are ineffective in PBC. It is hoped that a genetic explanation for the different patterns of disease presentation in PBC might be revealed and better targets for therapy still to be identified.

## Abbreviations

AIH: autoimmune hepatitis; ALP: alkaline phosphatise; AMA: antimitochondrial antibody; BCOADC: branched-chain 2 oxo-acid dehydrogenase complex; EVL: endoscopic variceal ligation; GGT: gamma-glutamyl transpeptidase; HCC: hepatocellular carcinoma; INR: international normalized ratio; MELD: model for end stage liver disease; MRCP: magnetic resonance cholangiopancreatography; NAFLD: non-alchoholic fatty liver disease; NRH: nodular regenerative hyperplasia; OGDC: 2-oxoglutarate dehydrogenase complex; OLT: orthotopic liver transplantation; PBC: primary biliary cirrhosis; PDC: pyruvate dehydrogenase complex; PDC-E2: E2 subunit of PDC; PSC: primary sclerosing cholangitis; PT: prothrombin time; SLE: Systemic lupus erythematosus; TCRBV3: T-cell receptor beta chain variable region; UDCA: Ursodeoxycholic acid; US: ultrasound
